# Supplementary figures and images for: Lipid kinases VPS34 and PIKfyve coordinate a phosphoinositide cascade to regulate retriever-mediated recycling on endosomes
Source: eLife. 2022 Jan 18;11:e69709. doi: 10.7554/eLife.69709 (PMC8816382; doi:10.7554/eLife.69709)

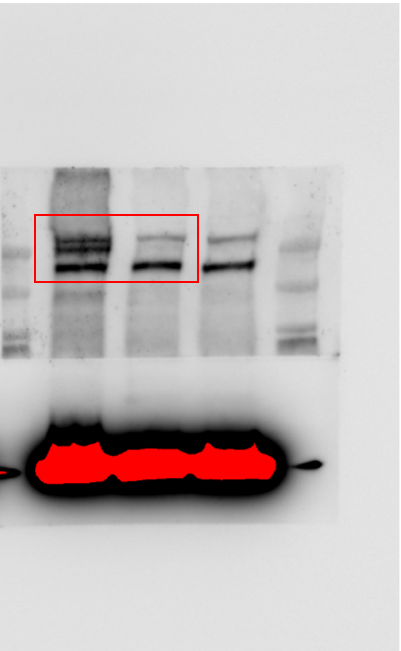

Supplement: Figure 1—figure supplement 3—source data 1. [file elife-69709-fig1-figsupp3-data1.zip › Figure 1-figure supplement 3- source data 1/Figure 1-figure supplement 3A Annotated source data1.tif]

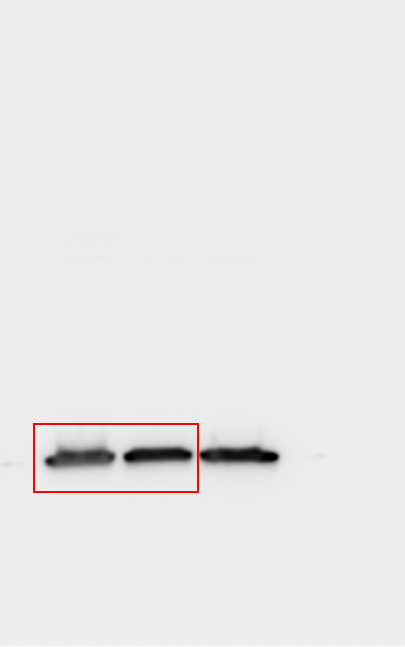

Supplement: Figure 1—figure supplement 3—source data 1. [file elife-69709-fig1-figsupp3-data1.zip › Figure 1-figure supplement 3- source data 1/Figure 1-figure supplement 3A Annotated source data2.tif]

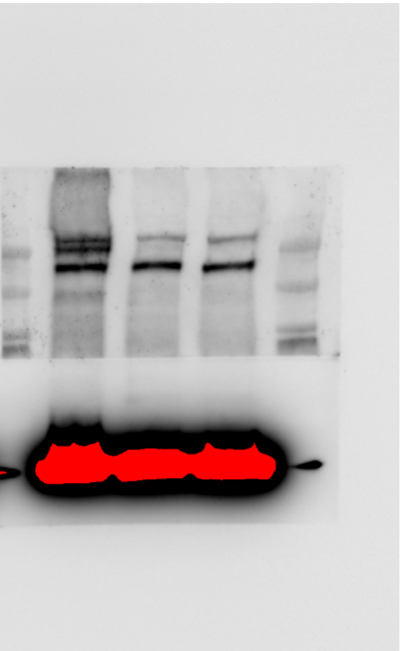

Supplement: Figure 1—figure supplement 3—source data 1. [file elife-69709-fig1-figsupp3-data1.zip › Figure 1-figure supplement 3- source data 1/Figure 1-figure supplement 3A Source data1.tif]

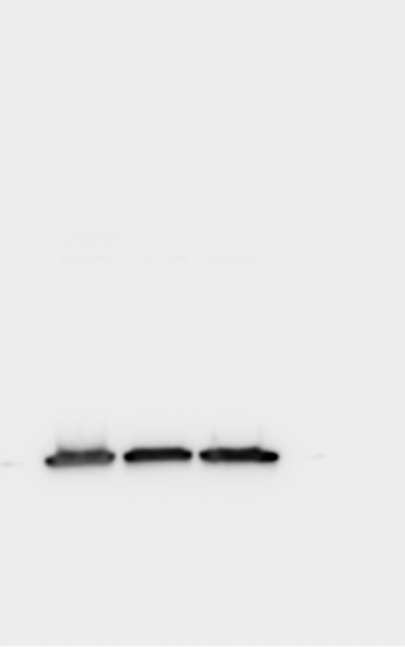

Supplement: Figure 1—figure supplement 3—source data 1. [file elife-69709-fig1-figsupp3-data1.zip › Figure 1-figure supplement 3- source data 1/Figure 1-figure supplement 3A Source data2.tif]

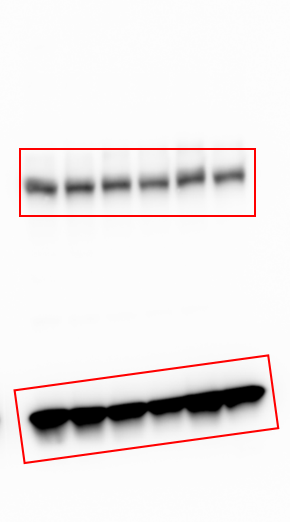

Supplement: Figure 5—figure supplement 1—source data 1. [file elife-69709-fig5-figsupp1-data1.zip › Figure 5-figure supplement 1- source data 1/Figure 5-figure supplement 1A-Annotated source data1.tif]

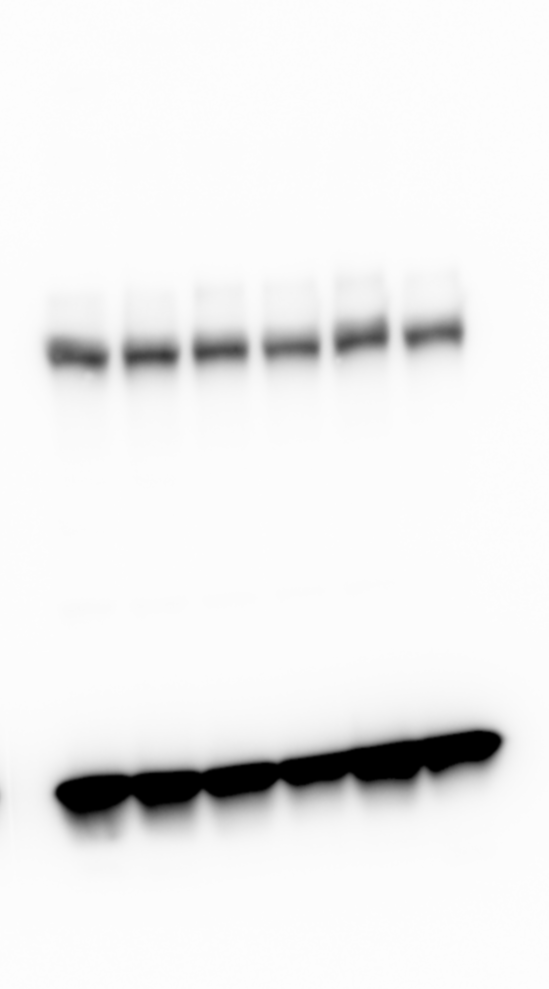

Supplement: Figure 5—figure supplement 1—source data 1. [file elife-69709-fig5-figsupp1-data1.zip › Figure 5-figure supplement 1- source data 1/Figure 5-figure supplement 1A-Source-Data1.tif]

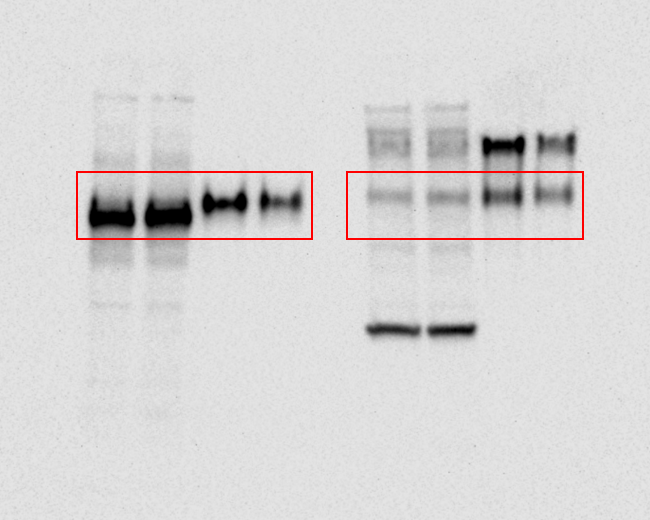

Supplement: Figure 6—source data 1. [file elife-69709-fig6-data1.zip › Figure 6-source data 1/Figure 6A-Annotated Source data1.tif]

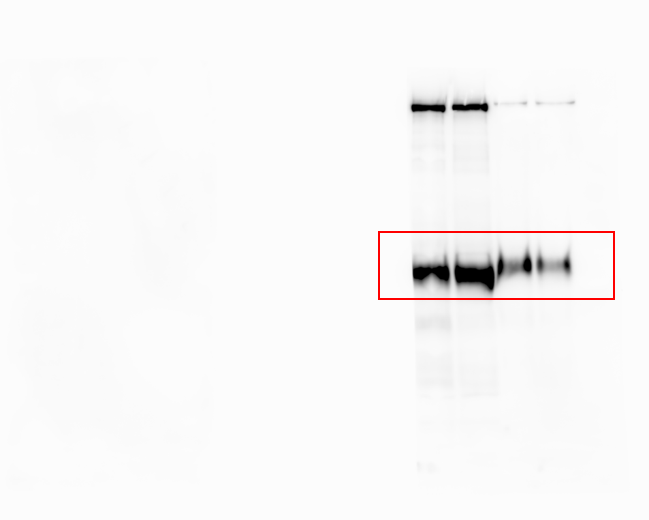

Supplement: Figure 6—source data 1. [file elife-69709-fig6-data1.zip › Figure 6-source data 1/Figure 6A-Annotated Source data2.tif]

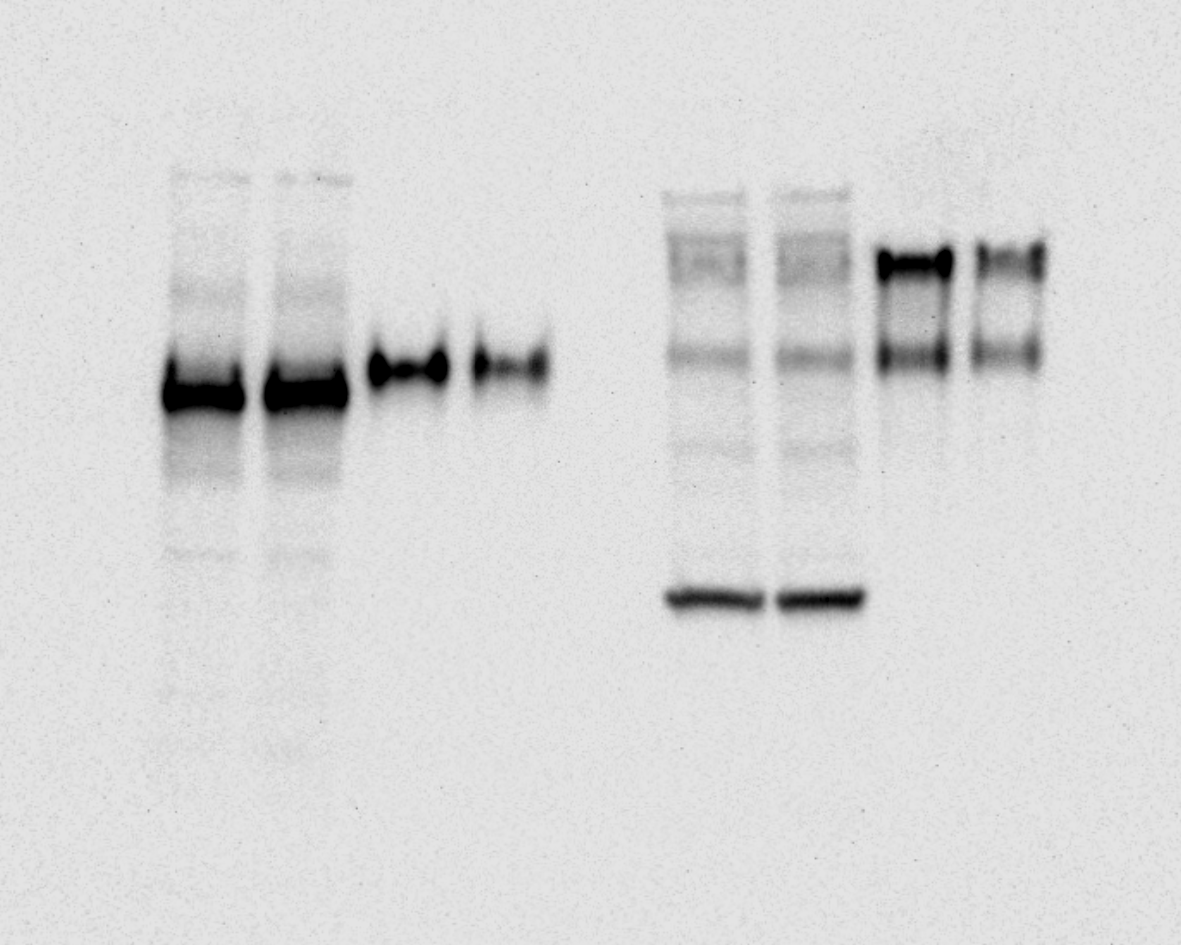

Supplement: Figure 6—source data 1. [file elife-69709-fig6-data1.zip › Figure 6-source data 1/Figure 6A-Source-Data1.tif]

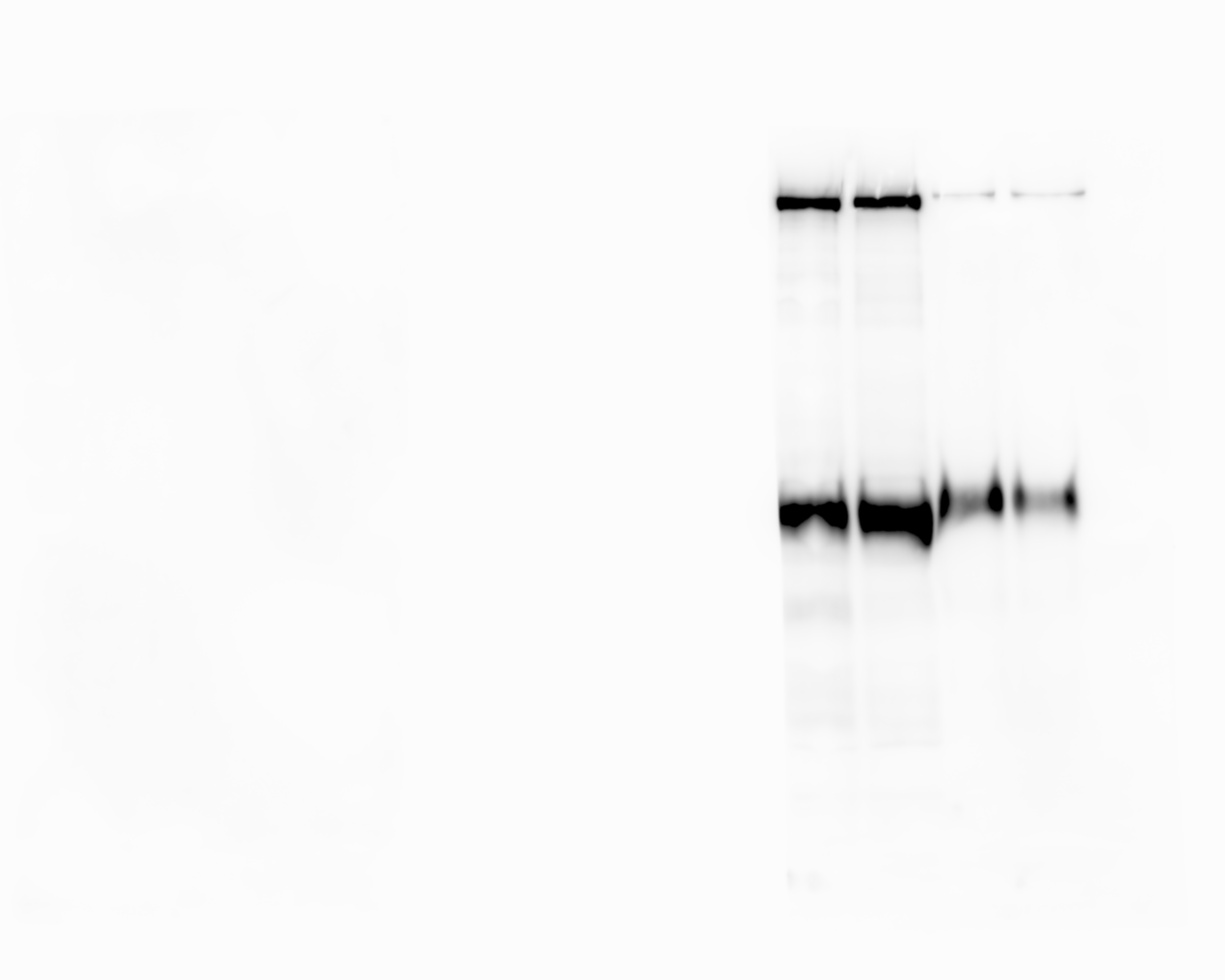

Supplement: Figure 6—source data 1. [file elife-69709-fig6-data1.zip › Figure 6-source data 1/Figure 6A-Source-Data2.tif]

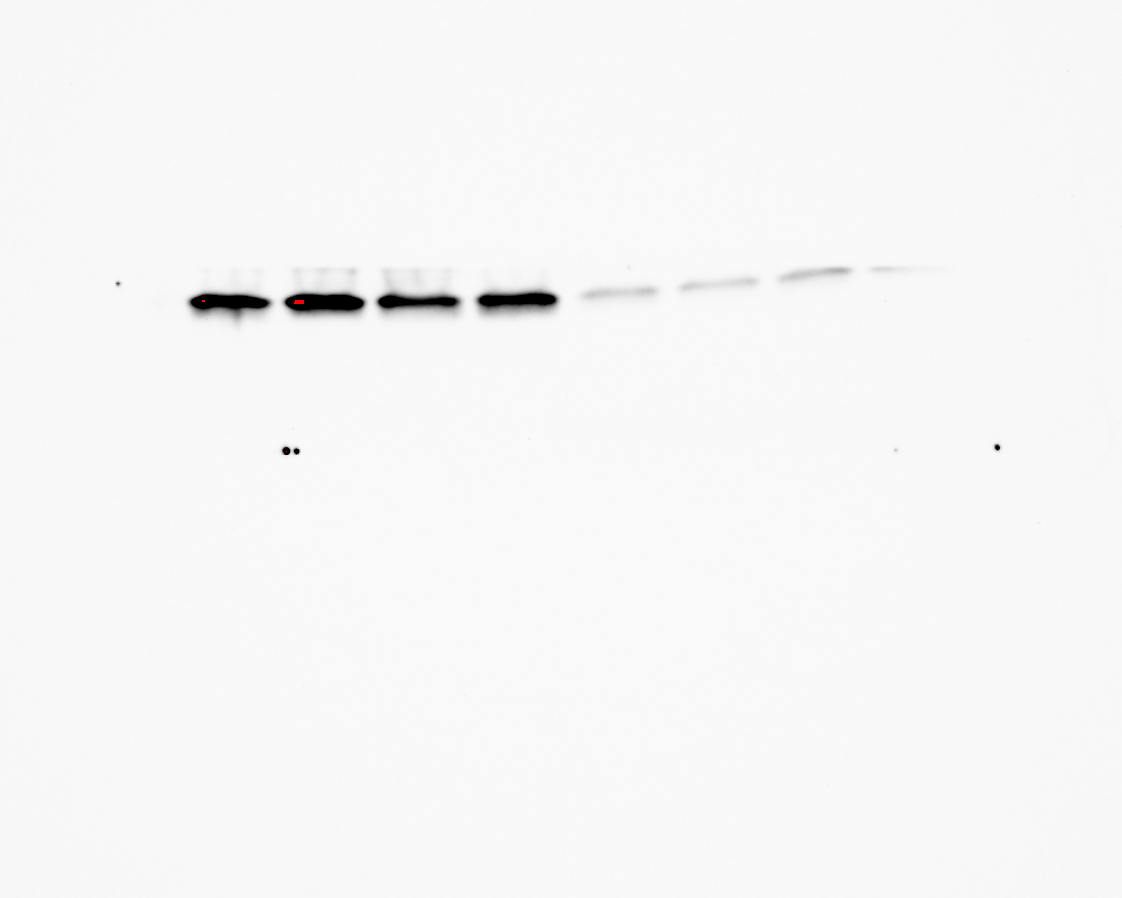

Supplement: Figure 7—figure supplement 6—source data 1. [file elife-69709-fig7-figsupp6-data1.zip › Figure 7-figure supplement 6- source data 1/Figure 7-figure supplement 6-Source data1.tif]

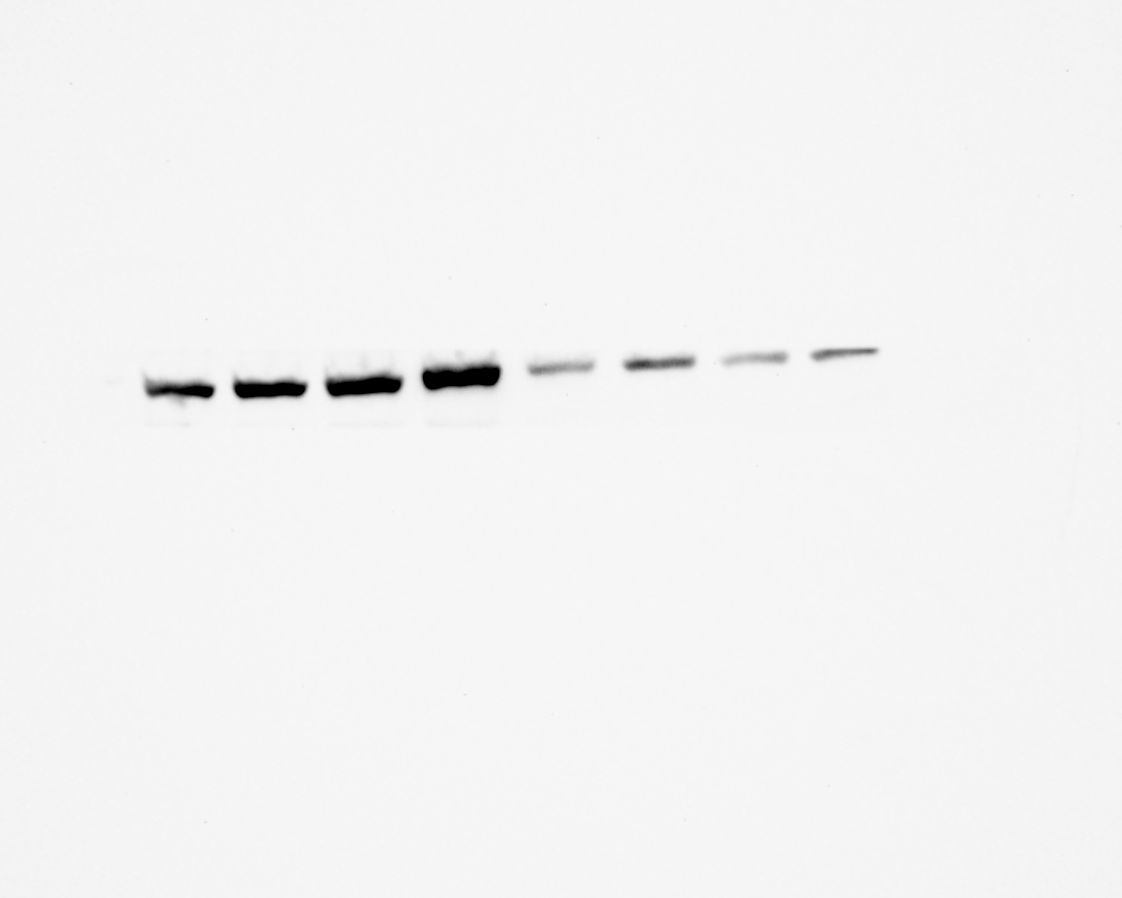

Supplement: Figure 7—figure supplement 6—source data 1. [file elife-69709-fig7-figsupp6-data1.zip › Figure 7-figure supplement 6- source data 1/Figure 7-figure supplement 6-Source data2.tif]

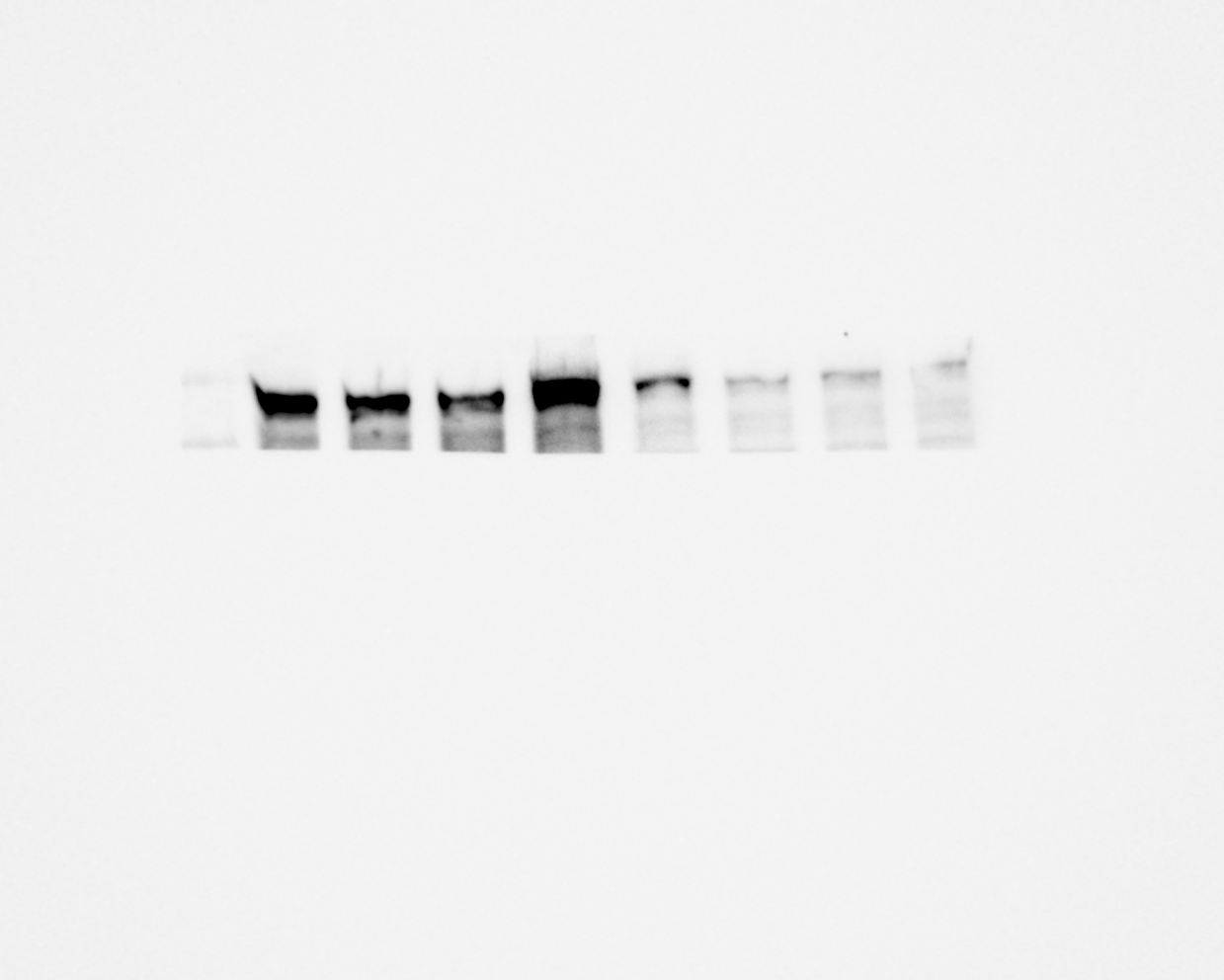

Supplement: Figure 7—figure supplement 6—source data 1. [file elife-69709-fig7-figsupp6-data1.zip › Figure 7-figure supplement 6- source data 1/Figure 7-figure supplement 6-Source data3.tif]

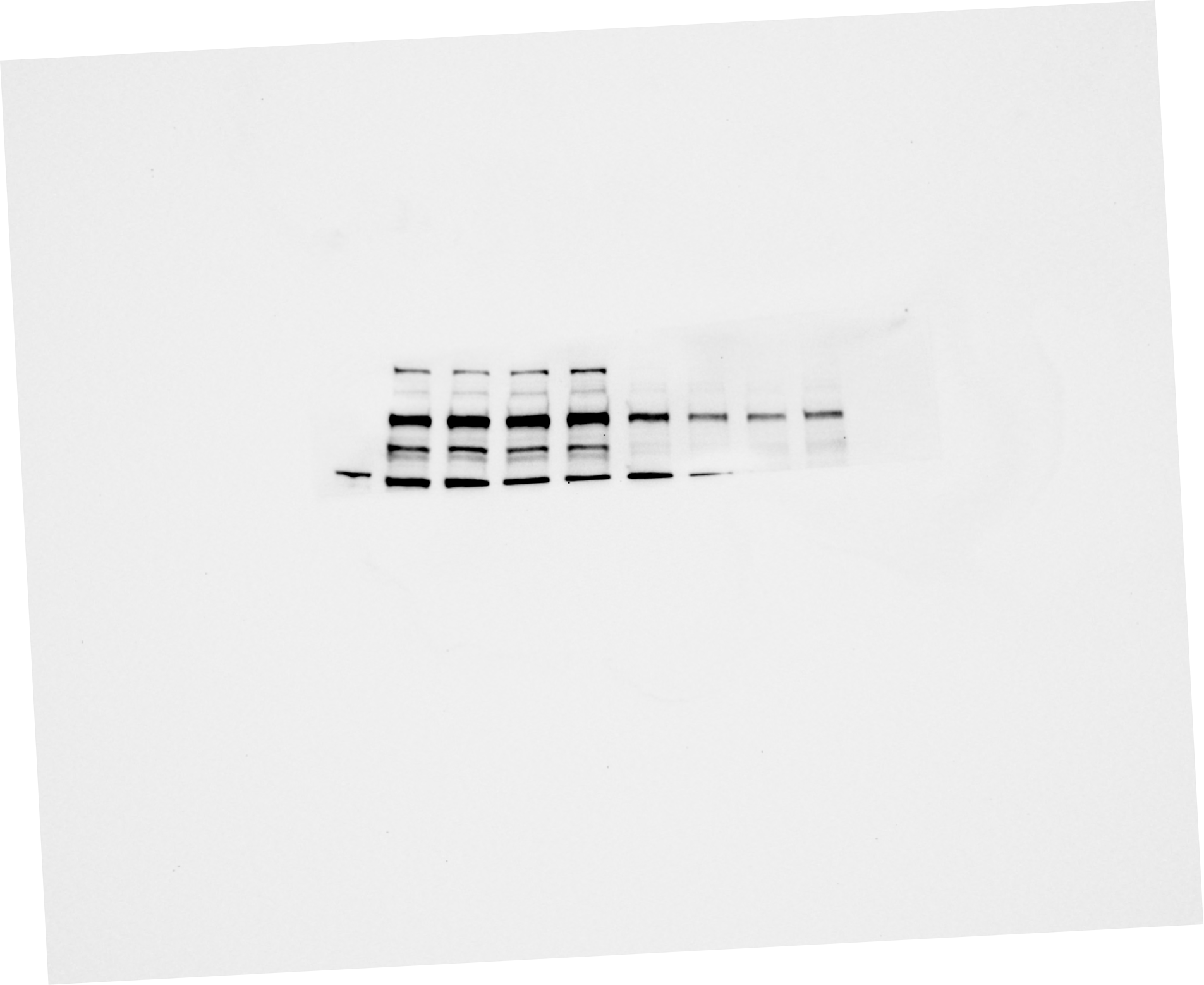

Supplement: Figure 7—figure supplement 6—source data 1. [file elife-69709-fig7-figsupp6-data1.zip › Figure 7-figure supplement 6- source data 1/Figure 7-figure supplement 6-Source data4.tif]

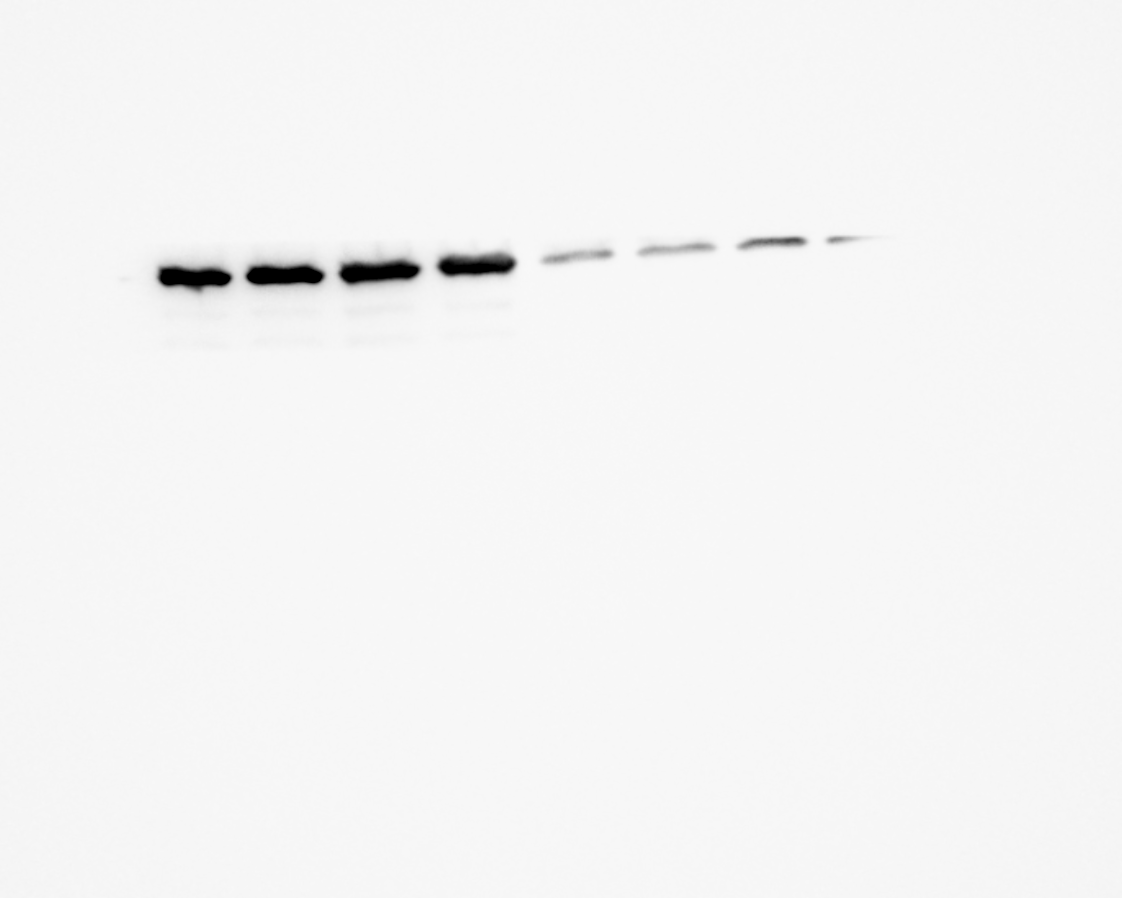

Supplement: Figure 7—figure supplement 6—source data 1. [file elife-69709-fig7-figsupp6-data1.zip › Figure 7-figure supplement 6- source data 1/Figure 7-figure supplement 6-Source data5.tif]

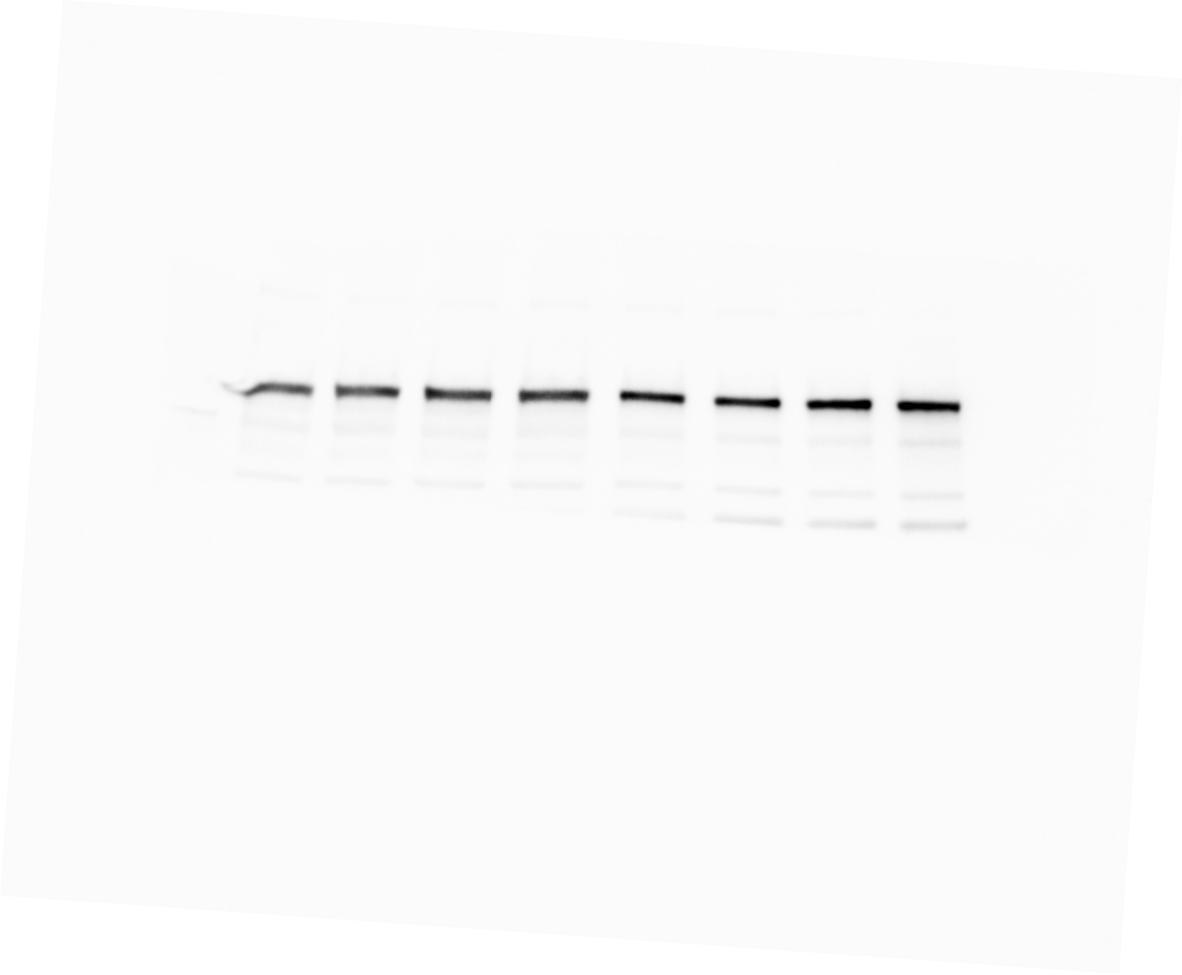

Supplement: Figure 7—figure supplement 6—source data 1. [file elife-69709-fig7-figsupp6-data1.zip › Figure 7-figure supplement 6- source data 1/Figure 7-figure supplement 6-Source data6.tif]

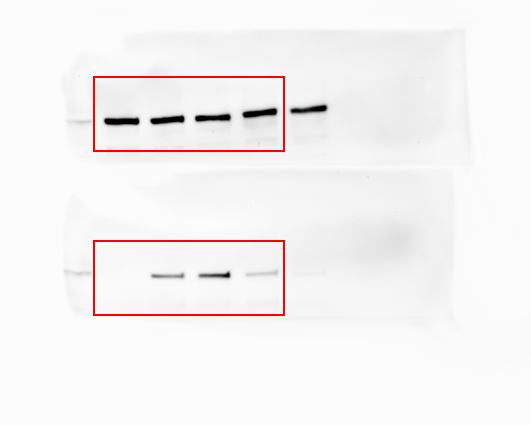

Supplement: Figure 9—figure supplement 1—source data 1. [file elife-69709-fig9-figsupp1-data1.zip › Figure 9-figure supplement 1- source data 1/Figure 9-figure supplement 1A-Annotated source data1.tif]

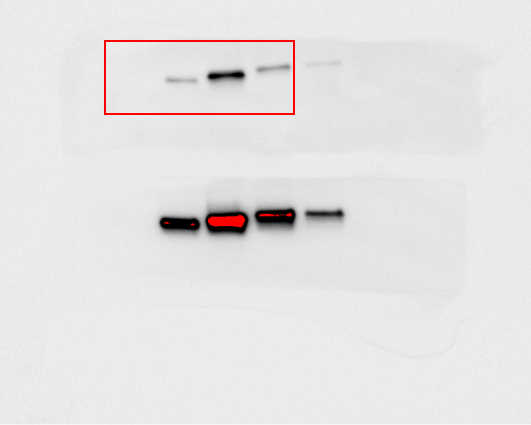

Supplement: Figure 9—figure supplement 1—source data 1. [file elife-69709-fig9-figsupp1-data1.zip › Figure 9-figure supplement 1- source data 1/Figure 9-figure supplement 1A-Annotated source data2.tif]

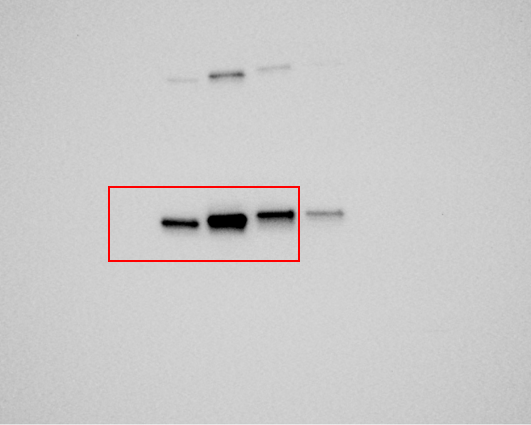

Supplement: Figure 9—figure supplement 1—source data 1. [file elife-69709-fig9-figsupp1-data1.zip › Figure 9-figure supplement 1- source data 1/Figure 9-figure supplement 1A-Annotated source data3.tif]

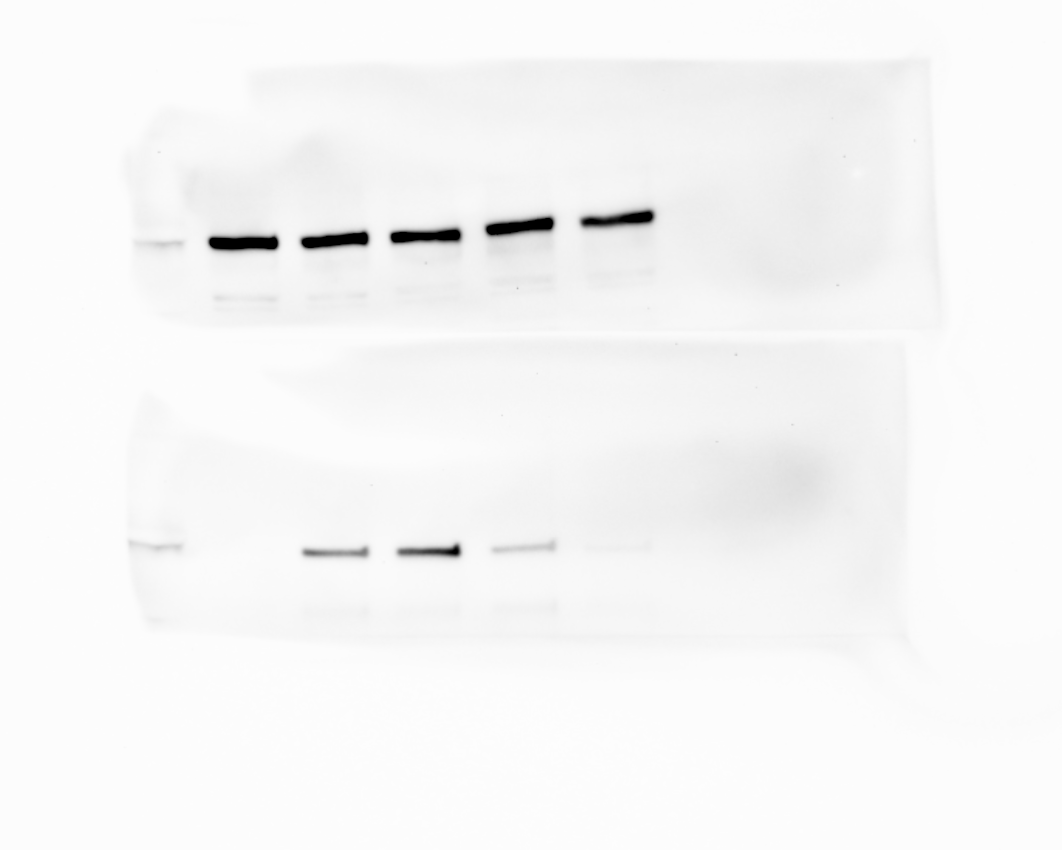

Supplement: Figure 9—figure supplement 1—source data 1. [file elife-69709-fig9-figsupp1-data1.zip › Figure 9-figure supplement 1- source data 1/Figure 9-figure supplement 1A-Source data1.tif]

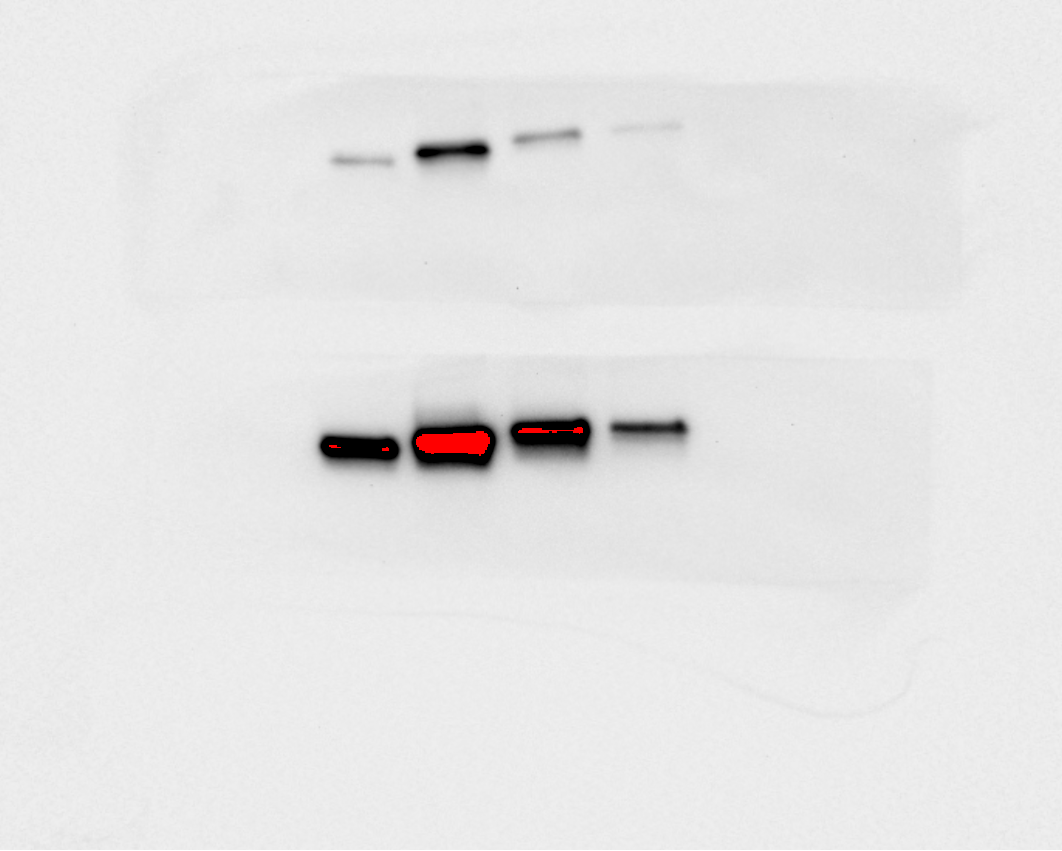

Supplement: Figure 9—figure supplement 1—source data 1. [file elife-69709-fig9-figsupp1-data1.zip › Figure 9-figure supplement 1- source data 1/Figure 9-figure supplement 1A-Source data2.tif]

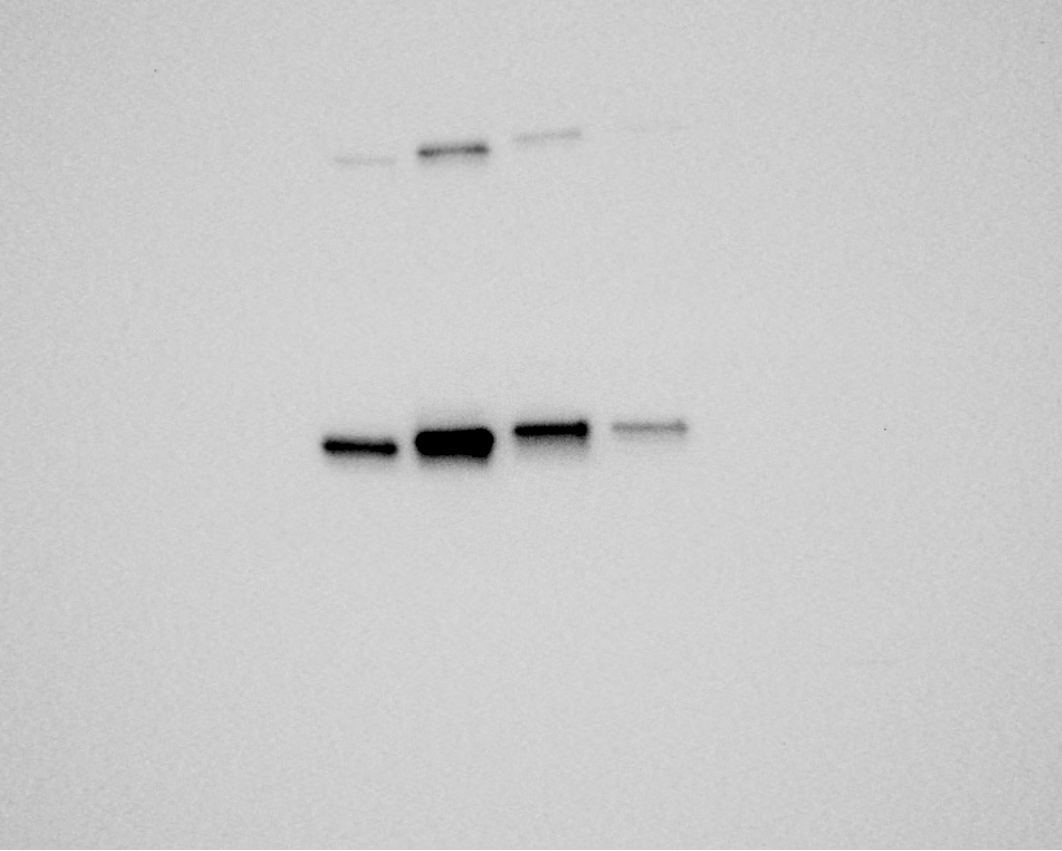

Supplement: Figure 9—figure supplement 1—source data 1. [file elife-69709-fig9-figsupp1-data1.zip › Figure 9-figure supplement 1- source data 1/Figure 9-figure supplement 1A-Source data3.tif]
